# Evolving HPV diagnostics: current practice and future frontiers

**DOI:** 10.3389/fcimb.2025.1681779

**Published:** 2025-11-11

**Authors:** Lin Shi, Haizhen Chen, Zichen Zhang, Yifei Wang, Wenbo Ren, Jing Huang

**Affiliations:** 1 Department of Clinical Laboratory, The First Hospital of Jilin University, Changchun, China; 2 College of Medical Technology, Beihua University, Jilin, China

**Keywords:** HPV detection, traditional methods, emerging technologies, real-time PCR, liquid biopsy, non-invasive screening

## Abstract

Human papillomavirus (HPV) infection serves as a primary causative agent of cervical cancer, highlighting the importance of early screening and detection in mitigating the incidence and mortality rates of HPV-related diseases. Over the past decades, HPV detection technologies have evolved considerably, transitioning from traditional methods to more advanced, patient-centered approaches. This review provides a comprehensive overview of both established and emerging HPV detection strategies, with a particular focus on their clinical applicability, technical advantages, and limitations. Conventional methods such as hybrid capture and PCR-based assays remain the backbone of clinical screening, offering robust sensitivity and specificity. However, their reliance on invasive sampling and centralized laboratory infrastructure limits accessibility and patient compliance, particularly in low-resource settings. To address these limitations, emerging technologies—including CRISPR/Cas systems, droplet digital PCR (ddPCR), next-generation sequencing (NGS), isothermal amplification techniques (IAT) and artificial intelligence (AI) combined with hpv screening offer enhanced accuracy, rapid turnaround, and the potential for point-of-care deployment. In parallel, innovations in sampling such as self-collected vaginal swabs and liquid biopsy using urine, blood, or extracellular vesicles are improving test acceptability and broadening screening coverage. By summarizing current progress and highlighting ongoing challenges, this review aims to guide the development of more precise, non-invasive, and scalable HPV detection strategies to reduce the global burden of HPV-related disease, support global prevention efforts, and guide public health policies.

## 1 Introduction

Human Papillomavirus (HPV), a small, non-enveloped, double-stranded DNA virus, encompasses over 200 distinct subtypes. Based on their carcinogenic potential, HPVs are classified into two broad categories: low-risk and high-risk types. Low-risk HPVs (such as HPV6 and 11) are typically associated with benign conditions like genital warts, whereas high-risk HPVs (hr-HPV) are closely linked to the development of various cancers (Godínez et al., 2014; Schiffman et al., 2016). Currently, the World Health Organization (WHO) has identified 12 hr-HPV types, including HPV16, 18, 31, 33, 35, 39, 45, 51, 52, 58, 59, and 68. Among these, HPV16 and 18 are the most common high-risk types, accounting for more than 70% of cervical cancer cases globally (Crosbie et al., 2013). These hr-HPVs are not only closely related to cervical cancer but also associated with multiple other malignancies such as anal cancer, oropharyngeal cancer, and penile cancer (Burd, 2003; Jensen et al., 2024), posing a significant threat to human health.

In recent decades, advancements in HPV screening technology and the widespread application of preventive measures such as vaccination have led to a gradual decline in the incidence of HPV infection and cervical cancer among women (Burd, 2016; Schiffman et al., 2016; Sundström and Elfström, 2020). For instance, the popularity of cytological screening (e.g., Pap smears) and HPV nucleic acid testing has significantly improved the detection rate of early lesions, enabling intervention and treatment of cervical cancer at the precancerous stage (Crosbie et al., 2013; Perkins et al., 2023). Furthermore, the rollout of HPV vaccines (such as bivalent, quadrivalent, and nonavalent vaccines), particularly with widespread coverage among adolescent girls, has effectively reduced the infection rate of hr-HPVs and the incidence of related cancers (Joura et al., 2015; Brisson et al., 2020).

Despite these achievements, HPV-related diseases remain a significant challenge in global public health, particularly in low- and middle-income countries where low screening and vaccination coverage have resulted in persistently high incidence and mortality rates of cervical cancer (Schiffman et al., 2016; Brisson et al., 2020). Therefore, further promoting HPV screening technology, increasing vaccination coverage, and developing more efficient and convenient detection methods and treatment strategies are crucial for comprehensively controlling HPV-related diseases and reducing the burden of cancer.

In the screening of HPV-related cancers, traditional cytological tests, such as the Pap smear or Liquid-based cytology (LBC), indirectly assess disease status by observing morphological changes in cells under a microscope. Despite being cost-effective and easy to implement, these methods have relatively low sensitivity and specificity, potentially leading to missed or misdiagnosed cases (Burd, 2003). In contrast, HPV nucleic acid testing can significantly enhance the accuracy and sensitivity of detection and has made substantial contributions to reducing the incidence and mortality rates of cervical cancer over the past decade (Ronco et al., 2014). Currently, the mainstream HPV molecular detection techniques in clinical practice primarily include two categories: nucleic acid hybridization signal amplification techniques, such as Hybrid Capture 2 (HC2), and nucleic acid amplification techniques and their derivatives, such as Polymerase Chain Reaction (PCR). These techniques primarily target HPV genomic DNA or mRNA, enabling direct detection of HPV infection and typing, thereby providing a more reliable basis for diagnosis (Williams et al., 2022).

**Figure 1:**
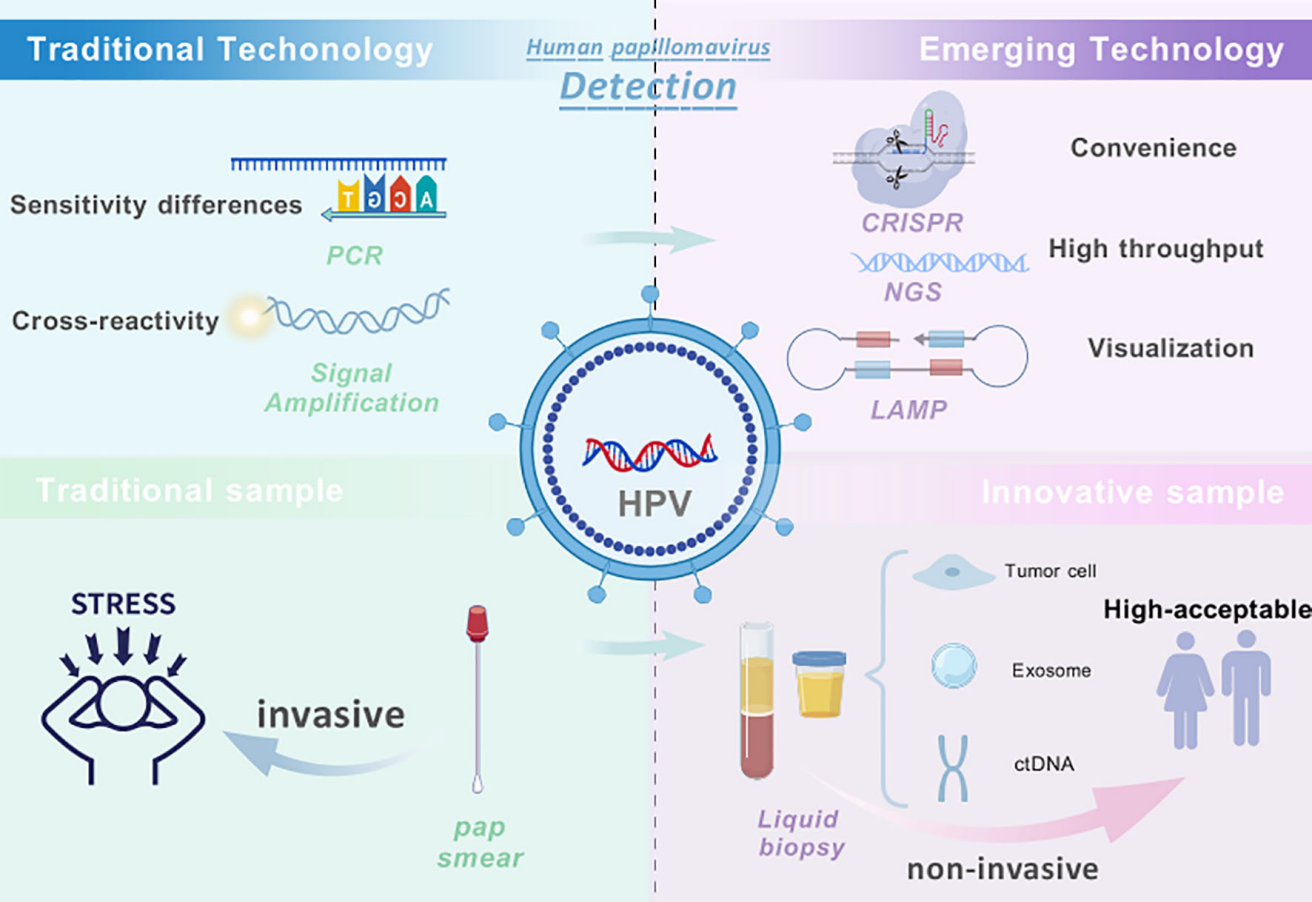
Overview of HPV detection technologies and sampling strategies. Traditional HPV detection methods, such as PCR-based DNA assays and signal amplification (e.g., HC2), have played a critical role in cervical cancer screening but rely on invasive cervical sampling and are limited by challenges such as cross-reactivity, insufficient genotyping, and accessibility in low-resource settings. In contrast, emerging technologies—including CRISPR-based assays, next-generation sequencing (NGS), and isothermal amplification techniques (IAT)—offer higher analytical sensitivity, faster detection, and potential for streamlined workflows. Meanwhile, innovations in sampling such as self-collected specimens and liquid biopsy using blood, urine, or exosome-derived material show promise in improving screening accessibility and patient compliance. Combining emerging technologies with non-invasive sampling approaches represents a forward-looking direction for developing more precise, patient-friendly, and scalable HPV detection strategies. Created with BioGDP.com ((Jiang et al., 2025).

In recent years, with the rapid advancement of molecular biology techniques, a variety of novel HPV detection technologies have been introduced into the clinical diagnosis and scientific research fields related to HPV. These include droplet digital PCR (ddPCR) (Mattox et al., 2022), next-generation sequencing (NGS) (Zhang et al., 2021), CRISPR-Cas12 (Zhan et al., 2023; Yin et al., 2024), nanotechnology (Avelino et al., 2021), and Isothermal Amplification Technology (IAT) (Rohatensky et al., 2018). In addition, building upon traditional methods, numerous studies are currently exploring innovations in sample types, sample collection methodologies, and detection approaches to develop more convenient and efficient HPV detection methods. Notably, the promotion and utilization of self-collected vaginal samples have significantly enhanced screening compliance, particularly in regions with limited medical resources (Gustavsson et al., 2018). Furthermore, detection methods utilizing urine, blood, or saliva based on extracellular vesicles (EVs) or cell-free nucleic acids (cfNAs) offer non-invasive and user-friendly screening options (Vorsters et al., 2012; Wang et al., 2020; Siravegna et al., 2022). These emerging detection technologies not only reduce the barriers to screening but also provide vital support for improving early diagnosis rates of HPV infections and decreasing cancer incidence (**Figure 1**).

According to our search, the most recent comprehensive review on HPV detection methods was published in 2022 (Williams et al., 2022), primarily focusing on clinically validated traditional detection methods. Despite the significant progress made by these methods in the field of HPV diagnosis, they still confront numerous challenges, such as inadequate sensitivity and specificity, high costs, and complex operational procedures. Therefore, the development and optimization of novel HPV detection methods to enhance their clinical practicality remain critical and challenging areas within the current field of HPV detection. This review aims to systematically summarize and analyze the current application status of HPV detection methods, and to classify them based on their clinical application scenarios and emerging technological trends. Notably, this review focuses primarily on studies and advancements reported after 2020, ensuring the inclusion of the most up-to-date evidence and developments. We will delve into the advantages and limitations of both traditional detection methods and emerging technologies, with a particular emphasis on the latest research advancements in non-invasive sampling techniques. Through in-depth analysis of existing technologies, this review seeks to provide a scientific basis for guiding the future direction of HPV detection research, thereby facilitating the formulation of more efficient early screening and prevention strategies for HPV-related cancers. Given the global health burden associated with HPV-related diseases, particularly in resource-limited areas, the publication of this review holds significant practical importance. It aims to provide theoretical guidance for the development of more convenient, economical, and efficient detection tools, thereby contributing to further advancements in global HPV prevention and control efforts.

## 2 Advances in HPV detection methods

Recent advances in HPV diagnostics have led to the development and evaluation of a broad spectrum of detection technologies. Clinically validated methods such as Hybrid Capture 2 (HC2), Cobas, BD Onclarity, and Aptima HPV (AHPV) remain widely used in screening programs due to their regulatory approval and robust sensitivity for high-risk HPV types. These assays, based on qPCR or transcription-mediated amplification, provide standardized workflows that facilitate large-scale cervical screening, particularly with clinician-collected cervical samples.

Meanwhile, the field is rapidly evolving with the introduction of emerging technologies aimed at improving detection accuracy, patient acceptability, and accessibility. Techniques such as next-generation sequencing (NGS), digital PCR (ddPCR), loop-mediated isothermal amplification (LAMP), and CRISPR-based diagnostics have been explored for their potential to provide high-throughput, low-cost, or point-of-care detection. Of particular interest are liquid biopsy approaches that utilize non-invasive samples, including blood, urine, and saliva, as well as extracellular vesicle (EV)-based assays that show promise in identifying HPV-derived nucleic acids and biomarkers. To provide a clearer comparison, **Table 1** categorizes detection technologies by their clinical maturity and scalability, while **Table 2** organizes sampling approaches by collection method and clinical applicability. (The main characteristics of the studies included in this review (e.g., study design, sample size, population demographics) and the key features of the HPV detection methods evaluated are summarized in **Tables 1**, **2**; **Supplementary Tables 1**, **2**).

**Table 1:** Advantages and disadvantages of the detection technologies included in the studies.

Category	HPV testing methods	Advantages	Critical challenges
Clinically Established Assays	HC2	∘ Higher sensitivity and reliability ∘ Can be used for mass screening	∘ Inability to differentiate between HPV subtypes ∘ Cross-reactivity
	Cervista	∘ Low sample size requirements ∘ Includes internal controls	∘ Inability to distinguish between specific subtypes ∘ Cross-reactivity
	Cobas	∘ High sensitivity ∘ Capable of detecting high-risk HPV types alone ∘ Clinically validated	∘ Higher price ∘ High equipment requirements ∘ May produce false positives
	BD Onclarity	∘ High sensitivity and specificity ∘ Recognises multiple HPV subtypes	∘ High cost ∘ False positives and false negatives risk
	PapilloCheck	∘ High sensitivity and specificity ∘ Recognises multiple HPV subtypes ∘ Accurate viral load quantification	∘ False positive risk ∘ High cost
	AHPV	∘ High specificity ∘ Can reflect the transcriptional activity of the virus ∘ Can assess cancer risk	∘ High cost ∘ Inability to distinguish between HPV subtypes ∘ Likelihood of false-negative results
	Abbott	∘ High sensitivity ∘ Rapid detection	∘ High cost ∘ Unable to provide virus load information ∘ False positive risk
Emerging Technologies with High-Throughput Potential	NGS	∘ High throughput ∘ Identifies multiple HPV subtypes and mutations ∘ Provides comprehensive genomic information	∘ High cost ∘ Specialized equipment
	ddPCR	∘ High sensitivity to detect low viral loads ∘ Absolute quantification, independent of standard curve ∘ High specificity	∘ Higher costs ∘ Requires specific equipment
Emerging Technologies with POCT Potential	LAMP/RPA	∘ Easy to operate and short duration ∘ No thermal cycler required ∘ Ideal for areas with limited resources	∘ Lower sensitivity vs. PCR ∘ complex primer design
	CRISPR/Cas12a	∘ High sensitivity, low cost ∘ Fast and easy to operate ∘ Visualisation of results ∘ Low detection limit (≤10 copies/μL)	∘ Higher requirements for purification of guide RNA and Cas12a protein ∘ Less demanding equipment but still needs to be optimised
	Nanotechnology	∘ High sensitivity and specificity ∘ Suitable for early screening ∘ No complex equipment required	∘ Technology is still in the development stage ∘ Low degree of standardisation of current equipment and operations

**Table 2:** Comparison of different sampling approaches and their compatible HPV detection platforms.

Sampling method	Detection target	Compatible detection techniques	Key advantages	Main limitations	Potential clinical application
Clinician-collected cervical samples	Exfoliated cellular HPV DNA/RNA	HC2, Cervista, PCR-based assays	High-quality standardized samples	Requires clinical setting	Routine cervical cancer screening
Self-collected samples (vaginal or cervical)	Exfoliated cellular HPV DNA/RNA	PCR-based assays, LAMP, CRISPR/Cas	Improves screening coverage; patient-friendly	Sampling variability; need for standardization	Home-based or low-resource screening programs
Liquid biopsy (urine, plasma, extracellular vesicles)	cfDNA, ctDNA, EV-derived HPV RNA/miRNA	PCR-based, ddPCR, NGS, CRISPR/Cas	Non-invasive, enables systemic or repeat monitoring, potential for vaccine/therapy evaluation	Low HPV DNA yield; requires high analytical sensitivity; complex isolation and analysis (EVs)	Early screening, treatment monitoring, vaccine efficacy assessment

Cumulatively, these technologies reflect a growing trend toward more personalized and less invasive HPV screening strategies. While clinical performance varies depending on sample type and detection principle, many novel approaches demonstrate comparable or superior sensitivity and specificity to traditional methods, suggesting strong potential for future clinical translation.

### 2.1 Traditional clinically validated assays

HPV molecular detection methods approved by regulatory bodies (e.g., FDA, Meijer criteria) are rigorously validated for clinical safety and efficacy. These technologies are categorized into signal amplification (e.g., HC2) and nucleic acid amplification platforms (e.g., PCR-based methods), which underpin current clinical practice in early screening and disease monitoring (**Table 3**) (Meijer et al., 2009; Arbyn et al., 2015; Fontham et al., 2020). While these traditional methods have established reliability, they face limitations in cost, subtype resolution, and invasiveness—challenges that emerging technologies aim to address. The following sections critically evaluate the strengths and weaknesses of these clinically validated approaches, setting the stage for discussing next-generation innovations.

**Table 3:** A summary of FDA-approved molecular diagnostic tests and clinically validated HPV detection methods in Europe.

Test	Technology type	Target	FDA approval or meets Meijer criteria (Meijer et al., 2009; Arbyn et al., 2015)
HC2	Signal Amplification	DNA	FDA and Meijer
Cervista	Signal Amplification	DNA	FDA and Meijer Partially validated
Cobas	Nucleic Acid Amplification	DNA	FDA and Meijer
BD Onclarity	Nucleic Acid Amplification	DNA	FDA
AHPV	Nucleic Acid Amplification	mRNA	FDA
Abbott	Nucleic Acid Amplification	DNA	Meijer
PapilloCheck	Microarray hybridization	DNA	Meijer

#### 2.1.1 Signal amplification

HC2 and Cervista are FDA-approved nucleic acid hybridization signal amplification technologies that detect HPV DNA directly in samples, without the need for PCR amplification. Specifically, HC2 and Cervista adopt distinct signal amplification strategies: chemiluminescence-based hybrid capture (HC2) vs. enzyme cleavage (Cervista) (Kurian et al., 2011; Burd, 2016).

##### 2.1.1.1 HC2

HC2, an FDA-approved HPV DNA assay, remains a cornerstone for large-scale cervical cancer screening. Utilizing chemiluminescent signal amplification via RNA-DNA hybridization (Clad et al., 2011; Xu et al., 2018), HC2 detects 13 hr-HPV types (HPV16, 18, 31, 33, 35, 39, 45, 51, 52, 56, 58, 59, 68) but lacks subtype differentiation (Wong et al., 2012; Williams et al., 2022).

Clinical trials demonstrate its high sensitivity (89–98%) for cervical intraepithelial neoplasia grade 2 and above (CIN2+) detection and reliability in large-scale screening, and its capable of detecting HPV infections at low abundance (Clad et al., 2011; Yu et al., 2015; Xu et al., 2018). Compared to cytological testing, HC2 can detect persistent high-grade cervical intraepithelial neoplasia (CIN) earlier, providing 60-70% greater protective efficacy in preventing invasive cervical cancer (Ronco et al., 2014).

However, HC2 lacks internal controls, leading to false negatives (5–12%) and cross-reactivity with non-target HPVs (e.g., HPV53, 61), resulting in false positives (Ratnam et al., 2010; Clad et al., 2011; Ronco et al., 2014). Park et al. indicates that compared to qPCR, HC2 exhibits higher clinical sensitivity for hr-HPV detection but has slightly lower specificity, and its inability to distinguish subtypes limits utility in genotype-guided management (Park et al., 2012).

##### 2.1.1.2 Cervista

Cervista employs Invader enzymatic signal amplification to detect 14 hr-HPV types (including the 13 types identified by HC2 and adding HPV66) using three probe pools (A5/A6, A7, A9), with the human histone 2 gene as an internalcontrol (Boers et al., 2014; Burd, 2016).

A cross-sectional clinical trial compared the clinical sensitivity (89% vs. 94%) and specificity (91% vs. 89%) of Cervista and HC2 in cervical cancer screening. Although Cervista has slightly lower sensitivity, it demonstrates good reliability and stability. Additionally, Cervista exhibits lower cross-reactivity with low-risk HPVs and requires only half the sample volume compared to HC2 (Kurian et al., 2011; Boers et al., 2014), which reduces the workload for sample collection and processing, and lowers testing costs. However, its sensitivity varies by HPV type: detection limits range from 1,250 copies/reaction for HPV16/18 to 7,500 copies/reaction for HPV35 (Burd, 2016). This variation in sensitivity suggests that, for certain HPV genotypes, combining Cervista with other diagnostic methods may be necessary to enhance the comprehensiveness and accuracy of the test.

#### 2.1.2 Nucleic acid amplification and derivatisation techniques

HPV nucleic acid amplification techniques primarily enrich specific DNA sequences within the HPV genome, either through PCR amplification or isothermal amplification. Following this enrichment, the sequences are detected using methods such as fluorescent probes, reverse dot blot hybridization, or gene chips.

##### 2.1.2.1 Cobas and BD Onclarity

Cobas HPV and BD Onclarity HPV are both based on qPCR technology, which amplifies target gene fragments during the detection process. These are currently among the most widely used HPV DNA detection methods (Harari et al., 2014). The main difference between the two is that Cobas HPV targets the highly conserved L1 gene sequence and uses β-globin as an internal control. It can detect the same 14 hr-HPV types as the Cervista and AHPV and can specifically genotype HPV-16 and HPV-18 (Wong et al., 2012). On the other hand, BD Onclarity focuses on detecting the E6/E7 oncogenes and provides typing information for six HPV types (HPV-16, 18, 31, 45, 51, and 52), along with detection results for three HPV genotype groups (HPV-33/58, HPV-35/39/68, and HPV-56/59/66) (Mesher et al., 2013; Bonde et al., 2020). In 2022, Boada et al. performed multiplex PCR testing on cervical cancer and precancerous lesion samples and found that the HPV DNA test results targeting the L1, E6, and E7 genes were highly consistent (Alcaniz Boada et al., 2023).

Clinical trial results indicate that Cobas test results show a high level of agreement with HC2 (up to 98%) (Wong et al., 2012; Mesher et al., 2013; Yu et al., 2015). The detection consistency between Onclarity and HC2 is 92% (Ejegod et al., 2016), and its overall consistency with GP-LMNX detection of hr-HPV is 93.6% (Bonde et al., 2020). The high agreement between both assays and traditional clinical assays is of significant reference value for clinical diagnosis, helping to reduce discrepancies in results caused by differences in detection techniques. Moreover, like other sensitive HPV detection methods, both Cobas and BD Onclarity effectively screen for high-risk patients in primary screening, with especially strong sensitivity for CIN3+ lesions (Mesher et al., 2013; Rebolj et al., 2014).However, despite their higher sensitivity in high-risk populations, both methods exhibit lower specificity for lesions below CIN2 (Cobas 4800 at 25.0%, BD HPV at 25.9%) (Mesher et al., 2013). This may lead to a certain number of false-positive results, thereby increasing the diagnostic burden and healthcare costs. Therefore, their application in the screening process should be considered comprehensively.

The Cobas HPV test offers quantitative detection capabilities, particularly for HPV16 and HPV18, which aids in assessing the persistence of infection and viral load. This information is vital for clinicians in determining whether further treatment and follow-up are needed (Yu et al., 2015). In contrast, the BD Onclarity focuses on the E6/E7 oncogenes, making it particularly sensitive in identifying HPV infections (Bonde et al., 2020). This assay outperforms tests targeting the L1 region, as the L1 region can be lost during the integration of viral DNA into the host’s genomic DNA, especially as the disease progresses to later stages. Therefore, tests targeting the L1 region may miss diagnosing advanced diseases (Pagliusi and Garland, 2007).

In summary, Cobas HPV offers advantages in monitoring infection persistence through quantitative L1 gene detection but may be limited by L1 loss in advanced disease. In contrast, BD Onclarity targets E6/E7 oncogenes for improved high-risk HPV identification, though it may face challenges in viral load assessment and genotyping complexity.

##### 2.1.2.2 Abbott

The Abbott RealTime HPV assay is an FDA-approved, PCR-based method utilizing real-time fluorescent quantitative PCR to target a conserved 150-bp region within the HPV L1 gene. It employs a modified GP5+/6+ primer system and four fluorescent probes to detect HPV16 and HPV18 (individually identified), 12 other high-risk HPV types (HPV31, 33, 35, 39, 45, 51, 52, 56, 58, 59, 66, 68) as a pooled result,and human β-globin as an internal control (Park et al., 2012; Mesher et al., 2013; Ducancelle et al., 2015).

This assay shows high consistency with the widely validated HC2 test, indicating its high reliability. Additionally, this test has very high sensitivity, enabling accurate detection and identification of HR-HPV infections (Wong et al., 2011). However, compared to other commonly used tests, it has a lower specificity for detecting High-grade Squamous Intraepithelial Lesion (HSIL/CIN2+) cases (Wong et al., 2011; Mesher et al., 2013), which may lead to unnecessary referrals and further testing, thus increasing healthcare costs and placing a psychological burden on patients. Unlike some other tests (e.g., ddPCR, Cobas), the Abbott HPV test does not provide quantitative information on HPV viral load (Mesher et al., 2013), which could be a limitation in assessing the severity of the infection and monitoring treatment outcomes.

##### 2.1.2.3 APTIMA

The AHPV assay is the only FDA-approved mRNA-based HPV test, utilizing quantitative reverse transcription PCR (qRT-PCR) to detect oncogenic E6/E7 mRNA transcripts from 14 hr-HPV types (including HPV16, 18, 45). Unlike DNA-based assays, AHPV directly assesses viral transcriptional activity, reflecting the integration of HPV into the host genome and carcinogenic potential (Clad et al., 2011; Iftner et al., 2015; Giorgi Rossi et al., 2022).

The AHPV test exhibits high specificity. In a diagnostic study by Clad et al., involving 451 women with abnormal cervical smear results, the AHPV test was compared with the HC2 test for CIN2+ detection. The sensitivity of the AHPV test was similar to that of the HC2 test (91.7% vs. 91.3%), but its specificity was significantly higher than that of HC2 (75.0% vs. 61.0%) (Clad et al., 2011). In other studies comparing AHPV with HPV DNA tests (HC2, Cobas4800), AHPV consistently demonstrated higher specificity in detecting CIN2+, with reduced cross-reactivity against non-target HPVs (Ratnam et al., 2011; Rebolj et al., 2014; White et al., 2024). The mechanistic advantage is that by targeting E6/E7 oncogenes, E6 protein degrades p53 via ubiquitination, disabling apoptosis and E7 protein inactivates pRb, driving uncontrolled cell proliferation, AHPV identifies active infections. This focus on transcriptional activity reduces false positives from transient HPV DNA presence, aligning results with precancerous progression risk (Iftner et al., 2015; Williams et al., 2022).

However, AHPV cannot differentiate HPV16/18/45 from other hr-HPV types, necessitating reflex genotyping for clinical decisions (Clad et al., 2011; Ratnam et al., 2011; Iftner et al., 2015). What’s more, mRNA testing may miss latent infections lacking E6/E7 expression, resulting in false negatives (Clad et al., 2011; Iftner et al., 2015). Additionally, mRNA extraction and testing are more complex than DNA testing, and mRNA is more prone to degradation, necessitating strict sample storage and handling conditions, and higher costs and equipment demands limit resource-limited adoption (Ratnam et al., 2011; Iftner et al., 2015).

#### 2.1.3 DNA microarray hybridization

The PapilloCheck HPV assay targets the conserved E1 open reading frame of HPV using multiplex PCR primers, amplifying a 350-bp region. Then amplified products hybridize with genotype-specific probes on a microarray chip. Fluorescent signals are analyzed to identify HPV types, covering 15 hrHPV: HPV16, 18, 31, 33, 35, 39, 45, 51, 52, 56, 58, 59, 68, 73, 82, 2 probable high-risk (pHR): HPV53, 66, 8 low-risk (lrHPV): HPV6, 11, 40, 42, 43, 44/55, 70 (Hesselink et al., 2010; Heard et al., 2016), which will support high-throughput genotyping and epidemiological studies. The PapilloCheck HPV assay demonstrates high sensitivity, detecting HPV at 100–500 copies/reaction, with 96.1% sensitivity for CIN2+ and 98.2% for CIN3+ in the Heard’s study (Heard et al., 2016). Its specificity reaches 91.6% in women aged ≥30 years; however, inclusion of additional subtypes (e.g., HPV73/82) reduces specificity by 5 to 8%, highlighting a trade-off between expanded genotyping and diagnostic precision (Hesselink et al., 2010).

Additionally, detection for certain HPV types (such as HPV-35 and 68) may exhibit lower consistency with other detection methods, and it may detect samples not identified by GP5/6-PCR-EIA, leading to a relatively high number of false positives (Hesselink et al., 2010; Schopp et al., 2010; Heard et al., 2016).

In conclusion, PapilloCheck excels in high-resolution HPV genotyping for research and screening but faces challenges in specificity and cost-effectiveness. Its clinical utility is validated under the Meijer criteria, yet optimization for low-resource settings remains critical.

Despite significant progress in improving HPV screening accuracy, current clinically validated methods such as HC2 and PCR-based assays (e.g., Cobas, Abbott, BD Onclarity) still face key limitations. HC2 lacks internal controls and genotyping capacity, increasing the risk of false positives/negatives and limiting its utility in precision screening (Ratnam et al., 2010; Clad et al., 2011; Wong et al., 2012). Similarly, while PCR-based methods offer high sensitivity, their specificity—particularly in detecting lesions below CIN2—is suboptimal, often leading to overdiagnosis and unnecessary clinical interventions (Wong et al., 2011; Mesher et al., 2013). Moreover, their reliance on invasive sampling and complex instrumentation reduces accessibility, especially in low-resource settings. These challenges underscore the need for next-generation HPV detection technologies that not only enhance analytical accuracy and genotyping resolution but also advance toward greater automation, throughput, and adaptability to diverse clinical and resource settings. In the following section, emerging molecular innovations—such as NGS, ddPCR, CRISPR-based platforms, and nanotechnology—are discussed for their potential to overcome these limitations and shape the future landscape of HPV diagnostics.

### 2.2 Emerging technologies

While next-generation HPV detection is envisioned to be accurate, simple, cost-effective, and patient-centered, achieving this balance often begins with high-precision innovations that drive future simplification. In this context, several emerging technologies including next-generation sequencing (NGS), digital PCR (dPCR), CRISPR-based assays, and nanotechnology are reshaping the analytical landscape. Although these methods are currently complex and resource-intensive, they provide the foundation for the next wave of miniaturized, automated, and low-cost point-of-care systems. The following subsections therefore present these technologies in two conceptual dimensions: (1) high-precision and high-throughput innovations that expand analytical capability, and (2) cost-efficient and field-deployable approaches that advance real-world accessibility.

#### 2.2.1 High-precision and high-throughput innovations

##### 2.2.1.1 NGS

NGS allows for a detailed analysis of the HPV genome, enabling the identification of various HPV types and their genetic variations (such as mutations, insertions, and deletions). This provides essential information regarding viral variability and pathogenicity (Zhang et al., 2021; Han et al., 2024).

The high sensitivity of NGS allows it to detect low-copy HPV infections and identify multiple infections, which is crucial for comprehensively assessing a patient’s infection status and disease risk. For instance, a study by Kathy Han and colleagues showed that seq-HPV, compared to dPCR (with a minimum detection level of 0.18 copies/mL), can sensitively detect HPV DNA from baseline plasma free DNA, identifying HPV DNA at as low as 0.023 copies/mL. Additionally, HPV ctDNA was detected in plasma from patients who tested negative in clinical tissue samples, suggesting that this could serve as an alternative to tumor tissue analysis (Han et al., 2024). In a study comparing NGS with Sanger sequencing for detecting HR-HPV, NGS demonstrated a sensitivity of 97.7%. Furthermore, 29 patients (25.7%) were found to have more HPV subtypes using NGS (Zhang et al., 2021). In another study by Andersen, which involved 93 paired samples, 25 (53.2%) HPV-positive samples from physician-collected specimens and 24 (54.5%) from self-collected samples showed multiple HPV type infections, further emphasizing NGS’s strong ability to detect co-infections (Andersen et al., 2022).

Moreover, NGS offers powerful genotyping capabilities, not only detecting the presence of HPV but also precisely identifying specific HPV subtypes. This is crucial for understanding the virus’s pathogenicity and disease progression. For example, Pasquier’s study utilized NGS-based PacBio single-molecule real-time sequencing (SMRT) technology to successfully identify multiple HPV types, such as HPV16, 18, and 31, and even detected multiple HPV genotypes in some samples (Pasquier et al., 2024). These features provide clinicians with more detailed information. NGS also has the ability to analyze E2 amplicons to determine whether HPV has integrated into the host genome, thus revealing the relationship between HPV and cancer (Andersen et al., 2022).

However, the high technical demands and complex data analysis involved in NGS limit its application in large-scale screening, and its detection costs are relatively high. Moreover, NGS cannot determine whether the virus is in an active infection state, making it more suitable for research and epidemiological studies rather than replacing transcriptional activity-based detection methods, such as the Aptima HPV test (Clad et al., 2011; Mesher et al., 2013; Iftner et al., 2015). Additionally, studies have shown that, compared to traditional PCR tests, which can produce results within a few hours, NGS generates a large volume of data that requires complex analysis, often taking 2–3 days to complete both detection and analysis (Zhang et al., 2021; Andersen et al., 2022; Han et al., 2024). Furthermore, some studies have noted that low DNA concentrations in samples may affect the accuracy of NGS detection (Andersen et al., 2022; Pasquier et al., 2024). In Pasquier’s study, tissue fixation led to DNA fragmentation, which affected the results, meaning that long-read strategies are not suitable for paraffin-embedded or formalin-fixed samples (Pasquier et al., 2024).

Despite its current limitations in cost, turnaround time, and data complexity, NGS holds significant promise for the future of HPV diagnostics. With continuous reductions in sequencing costs and the development of streamlined library preparation and bioinformatic pipelines, NGS is expected to become more accessible for clinical use. In particular, the integration of artificial intelligence (AI) and machine learning algorithms may enable automated interpretation of large-scale HPV genomic data, improving accuracy in genotype identification, viral integration mapping, and mutation profiling (Tian et al., 2021). Furthermore, combining NGS with liquid biopsy platforms—such as circulating cfDNA(cell-free DNA) or extracellular vesicle-derived HPV transcripts—could enable dynamic monitoring of viral evolution, treatment response, and vaccine efficacy through minimally invasive sampling. To translate these applications into clinical practice, large-scale prospective studies and standardized analytical frameworks are needed to validate the diagnostic and prognostic utility of NGS-based HPV assays.

##### 2.2.1.2 ddPCR

Droplet digital PCR (ddPCR) partitions the PCR reaction mixture into 20,000 nanoliter-sized droplets, each containing 0–1 target DNA molecules. Post-amplification, droplets are classified as positive (fluorescent signal) or negative, enabling absolute quantification of HPV DNA/RNA via Poisson distribution analysis (Mattox et al., 2022; Siravegna et al., 2022). This approach eliminates reliance on standard curves, providing unparalleled precision in low-copy detection.

ddPCR offers several advantages for HPV detection, including ultra-sensitive detection, which allows for the identification of HPV16 in plasma with 69.8% sensitivity for oropharyngeal cancer (OPC), outperforming qPCR (20.6%) (Mattox et al., 2022). It can also detect HPV16 at 1.6 copies/μL in FFPE and liquid-based cytology (LBC) samples (Lillsunde Larsson and Helenius, 2017). Its quantitative accuracy is exceptional, with a lower limit of detection (LLOD) ranging from 0.08–0.5 copies/μL for HPV31/35/39/51/56 (Malin et al., 2021), enabling early-stage infection monitoring. The method offers absolute quantification, directly measuring HPV DNA copies without the need for standard curves, which is crucial for assessing viral load dynamics during treatment (Siravegna et al., 2022). Additionally, ddPCR is versatile, compatible with various sample types (blood, FFPE, LBC), making it suitable for both retrospective and prospective studies (Lillsunde Larsson and Helenius, 2017).

However, there are limitations to ddPCR. The cost and complexity of the method are significant drawbacks, as it requires specialized equipment (e.g., QX200 system) and reagents, increasing per-test costs (Lillsunde Larsson and Helenius, 2017; Malin et al., 2021). Additionally, the procedural complexity demands skilled operators, which limits scalability in resource-limited settings. ddPCR is also primarily optimized for single-target detection, and simultaneous genotyping of multiple HPV types requires sequential assays (Mattox et al., 2022). The throughput is another constraint, with a lower sample capacity (96 samples per run) compared to NGS or microarray platforms (Han et al., 2024).

Looking ahead, the combination of ddPCR with liquid biopsy and cfDNA analysis promises a highly sensitive and noninvasive approach for HPV detection. This strategy could be pivotal in detecting minimal residual disease and dynamically monitoring treatment response or vaccine efficacy. To achieve broader clinical translation, future research should focus on reducing costs through microfluidic miniaturization, enhancing multiplexing capability for simultaneous HPV genotyping, and standardizing pre-analytical workflows to ensure reproducibility across laboratories. Ultimately, ddPCR could evolve into a powerful precision tool for real-time surveillance of HPV-driven cancers and personalized therapeutic monitoring.

##### 2.2.1.3 Nanotechnology

Nanotechnology for HPV detection leverages the optical, electrical, and magnetic properties of nanomaterials such as nanoparticles, nanosensors, and nanostructures. Techniques like Surface-Enhanced Raman Scattering (SERS), Localized Surface Plasmon Resonance (LSPR), and nanobiosensors are utilized to achieve precise HPV detection by capturing the signal changes resulting from the specific binding of nanomaterials to HPV molecules (Mao et al., 2017; Avelino et al., 2021; Zhan et al., 2023).

Nanotechnology exhibits unique advantages in HPV detection, primarily in terms of high sensitivity, excellent specificity, relatively simple operation, rapid detection speed, and multiplexing capabilities. For instance, research by Mao et al. (2017) demonstrated that the Cys-Sso7d/Au NP probe, in combination with PCR, can detect as few as 1 copy of the HPV gene, outperforming traditional PCR gel electrophoresis with a detection limit of 10 to 10² copies. Additionally, this probe exhibits detection specificities of 100.0% and 91.7% for HPV 16 and HPV 18, respectively, in clinical Pap smear specimens, thanks to the specific DNA-binding properties of the Sso7d protein. Furthermore, compared to traditional PCR techniques, this detection method simplifies operational steps, shortens detection time, and in some studies, the entire detection process takes only 15 minutes (Avelino et al., 2021), facilitating rapid diagnosis. It is also noteworthy that Zhan et al.’s research, through the combination of DNA tetrahedrons, the CRISPR-Cas12a system, and Inductively Coupled Plasma Mass Spectrometry (ICP-MS), achieved simultaneous detection of multiple HPV-DNA genotypes such as HPV-16, HPV-18, and HPV-52, significantly enhancing detection efficiency (Zhan et al., 2023).

However, nanotechnology also exhibits certain limitations in HPV detection. While some nanotechnology-based detection methods claim relative simplicity, others still involve intricate experimental procedures and costly instrumentation, potentially impeding their widespread adoption in primary healthcare settings. Additionally, these methods may have stringent requirements for sample pretreatment; improper handling of samples can result in biased detection results (Mao et al., 2017; Avelino et al., 2021; Zhan et al., 2023). Therefore, further research and optimization of sample pretreatment methods are necessary when promoting nanotechnology for HPV detection. Considerations must also be given to reducing technical complexity and costs to facilitate broader application in clinical practice.

In summary, nanotechnology demonstrates high sensitivity, excellent specificity, and rapid detection capabilities in HPV testing, enabling more precise and swift virus identification. Nevertheless, challenges persist in its promotion due to high equipment requirements, complex operations, elevated costs, and limited related research. Looking ahead, the integration of nanotechnology with other molecular diagnostic innovations—such as CRISPR/Cas systems, artificial intelligence, and liquid biopsy—could drive the development of next-generation HPV testing platforms with enhanced automation and real-time analytics. Future research should focus on improving nanoparticle stability, miniaturizing detection devices for point-of-care testing (POCT), and conducting large-scale clinical validation to ensure translational feasibility. Such efforts will be key to transforming nanotechnology-based HPV diagnostics from laboratory innovation into clinically accessible tools for early cancer screening and monitoring.

##### 2.2.1.4 CRISPR-based HPV detection

The evolution of CRISPR technology from Cas9 to Cas12 and Cas13 reflects a transition from precise DNA recognition to collateral cleavage-mediated signal amplification and RNA-level detection, expanding its diagnostic potential across diverse viral systems.This evolution reflects a broader shift toward rapid, sensitive, and multiplex HPV diagnostics suited for both centralized and POCT.

Cas9-based assays established the foundation of CRISPR diagnostics through their precise, sequence-specific DNA recognition guided by single-guide RNAs (sgRNAs). Early systems such as CRISPR-typing PCR (ctPCR) and Cas9/sgRNA-associated reverse PCR (CARP) demonstrated accurate genotyping of high-risk HPV types, achieving detection limits down to the femtomolar range with no cross-reactivity (Zhang et al., 2018). Su et al. and Zhang et al.’s subsequent innovations enhanced detection versatility by integrating Cas9 or dCas9 with biosensing platforms, including surface-enhanced Raman scattering (SERS) and hyperbranched rolling circle amplification (HRCA), which further improved sensitivity and enabled visual or electrochemical readouts (Zhang et al., 2022; Su et al., 2023). These advances expanded Cas9’s potential beyond PCR-based workflows, offering high analytical precision and compatibility with diverse detection formats. Cas9-based systems offer high structural precision and serve as the conceptual foundation for later Cas12- and Cas13-based diagnostic platforms. However, unlike Cas12 or Cas13, Cas9 lacks collateral cleavage activity, which limits intrinsic signal amplification (Di Carlo and Sorrentino, 2024). Consequently, most Cas9 assays still depend on multi-step workflows and pre-amplified DNA templates, often requiring thermocycling or laboratory-grade instruments. These constraints reduce their suitability for true POCT applications, although their robust target recognition continues to underpin the evolution of next-generation CRISPR diagnostics.

Building upon the sequence-specific cleavage mechanism of Cas9, the CRISPR-Cas12 system introduces a collateral cleavage capability that enables sensitive and rapid detection of HPV DNA. Guided by a specific crRNA, Cas12a recognizes target sequences within HPV oncogenes such as E6 and E7, and upon activation, nonspecifically cleaves single-stranded DNA reporters, generating fluorescence or colorimetric signals. This mechanism supports simple, highly sensitive assays capable of detecting as few as 1–2 copies/μL of high-risk HPV DNA within 30 minutes, without the need for thermocyclers or complex instrumentation (Liu et al., 2024; Yin et al., 2024). Recent studies have demonstrated that Cas12-based platforms can accurately identify up to 14 hr-HPV types with high specificity, while maintaining compatibility with multiplexed and visual detection formats (Zhan et al., 2023; Yin et al., 2024).When integrated with isothermal amplification methods such as RPA, Cas12-based assays achieve rapid and field-deployable performance. For instance, Liu et al. validated an RPA–Cas12a assay on 258 cervical swab samples, achieving 100% sensitivity and specificity within a single-tube reaction (Liu et al., 2024).

Beyond laboratory settings, the minimal equipment requirements and visual readout make Cas12 particularly attractive for POCT. Its potential to deliver low-cost, rapid, and accurate HPV detection aligns with the growing demand for decentralized cervical cancer screening, especially in resource-limited regions. However, technical refinements such as gRNA design optimization, reagent stability, and cost reduction remain necessary to ensure consistent performance in clinical and field environments. Overall, CRISPR-Cas12 represents a major step forward in HPV diagnostics, bridging laboratory precision with the accessibility essential for next-generation POCT platforms.

Following the DNA-targeting capabilities of Cas9 and the collateral cleavage amplification of Cas12, the Cas13 system extends CRISPR diagnostics to the RNA level, enabling direct detection of transcriptionally active HPV infections. Cas13 effectors, guided by crRNA, specifically recognize HPV RNA sequences such as E6/E7 transcripts and activate non-specific RNase activity that cleaves labeled RNA reporters to produce a detectable signal (He et al., 2025). This RNA-based readout provides functional information on viral oncogene expression, offering a potential advantage in distinguishing clinically relevant infections from transient HPV DNA carriage. Recent studies have demonstrated the versatility of Cas13-based assays in multiplex and POCT. Zheng et al. developed a Cas13a/Cas12a dual-channel platform capable of simultaneously detecting HPV16 and HPV18 with high sensitivity (limit of detection ≈10 copies per reaction) and visual readout within 40 minutes (Zheng et al., 2022), while Ghouneimy et al. established a one-pot multiplex CRISPR platform integrating Cas12 and Cas13 for rapid and accurate detection of multiple hr-HPV genotypes with excellent specificity (Ghouneimy et al., 2024). Cas13 extends HPV diagnostics to the RNA level, enabling early detection of active infections and monitoring of viral gene expression. Compared with Cas12 systems, it offers transcriptional insights, supports multiplexing, and is compatible with portable fluorescence or lateral-flow devices, enhancing POCT adaptability. However, RNA instability and the need for precise crRNA design remain technical challenges. Overall, Cas13 complements DNA-based assays by adding transcriptional depth and expanding CRISPR’s potential for rapid, multiplex HPV detection in diverse clinical settings.

Although CRISPR-based HPV detection has shown great promise, its clinical translation remains at an early stage. Current studies are largely laboratory-based, and several challenges must be addressed before it can be applied to POCT. These include optimizing specimen sources for easier, noninvasive sampling, expanding detection coverage to accurately distinguish genotypes with high sequence similarity, and improving reagent stability for room-temperature operation. Moreover, the complexity of multiple HPV infections requires assays capable of multiplex and quantitative analysis using minimal sample input. Simplifying reaction steps and reducing dependence on nucleic acid amplification will further enhance speed and minimize contamination risk (Ghouneimy et al., 2022; Wang et al., 2025). Future efforts should focus on enzyme engineering, integrated microfluidic platforms, and portable readout systems to improve robustness and usability. With continued optimization, CRISPR-based assays are expected to evolve into efficient, low-cost, and field-deployable diagnostic tools for HPV screening, especially in resource-limited settings.

#### 2.2.2 Cost-efficient and point-of-care-oriented approaches

##### 2.2.2.1 IAT

IAT is an emerging method in molecular diagnostics that has been increasingly applied in recent years. The main feature of IAT is the ability to amplify DNA or RNA at a constant temperature, eliminating the need for thermal cycling. Compared to traditional PCR technology, IAT is simpler to operate and requires less specialized equipment, offering significant advantages for clinical diagnostics, field testing, and use in resource-limited settings. Several common isothermal amplification techniques are used for HPV detection, including:

##### 2.2.2.2 LAMP

LAMP technology, an innovative molecular diagnostic tool, has shown significant advantages in HPV detection. It amplifies DNA efficiently using specific primers and DNA polymerase at a constant temperature (around 65 °C), generating large amounts of target DNA products. The results can be visually detected through changes in color or turbidity (Rohatensky et al., 2018; Vo and Story, 2021).

The high specificity of LAMP enables accurate differentiation among HPV subtypes. In a study by Rohatensky et al., subtype-specific LAMP assays successfully amplified HPV 16, 18, 31, and 35 from high-concentration plasmid templates (10^6^ copies) without cross-amplification (Rohatensky et al., 2018). The same study also showed that LAMP reactions could be performed directly on heat-treated cellular lysates without DNA purification, highlighting its simplicity and robustness. Similarly, Izadi et al. developed an electrochemical LAMP bioassay capable of detecting HPV DNA directly from crude lysates, achieving a sensitivity sufficient to detect viral DNA from as few as 10 cells (Izadi et al., 2021). These findings demonstrate that LAMP can substantially streamline the detection process, minimize handling steps, and provide results within 30 minutes, making it particularly suitable for primary healthcare and resource-limited environments.

However, LAMP performance may vary depending on viral subtype, target molecule, and sample type. In a plasmid-based study, Rohatensky et al. reported subtype-specific detection limits of approximately 10^5^ copies for HPV16, 10³ copies for HPV18, 10^4^ copies for HPV31, and 10^5^ copies for HPV35 (Rohatensky et al., 2018). In addition, in a more recent investigation, Izadi et al. (2024) designed a LAMP-based electrochemical platform to assess HPV16 genome integration at the mRNA level by comparing E7 and E2 transcript expression in cervical cancer samples. This assay focused on monitoring viral integration status, and demonstrated strong concordance with qRT-PCR, ddPCR, and immunohistochemistry (Izadi et al., 2024).

Because these studies differ in analytical targets (DNA vs. mRNA), experimental models (plasmid, cell lysate, or clinical tissue), and intended applications (subtype identification vs. integration monitoring), their sensitivity values are not directly comparable. Collectively, however, they illustrate the remarkable versatility of LAMP—spanning rapid HPV subtype detection, direct analysis from minimally processed samples, and integration-status monitoring. Further optimization and clinical validation are needed to standardize LAMP performance across diverse HPV genotypes and sample types, advancing its translation toward routine clinical diagnostics.

##### 2.2.2.3 Other isothermal amplification assays

In addition to LAMP, several other isothermal amplification assays such as Recombinase Polymerase Amplification (RPA) and AmpFire (Amplification by Firefly)—have emerged as promising tools for HPV detection. RPA performs DNA amplification at lower temperatures (typically 37–42°C) through the coordinated activity of recombinase enzymes, single-stranded binding proteins, and strand-displacing polymerases. Amplification can be completed within 15–30 minutes, and the products can be detected through fluorescence or lateral flow assays. Studies have shown that RPA achieves analytical sensitivity and specificity comparable to conventional PCR, detecting as few as 1–10 copies per reaction (Liu et al., 2024). Moreover, it has been successfully applied in multiplexed HPV typing assays when coupled with CRISPR/Cas-based readouts, enabling POCT-level HPV genotyping with minimal thermal control (Flores-Contreras et al., 2024). Its rapid kinetics and portability make it an attractive candidate for HPV screening in community-based or field environments.

The AmpFire system, in contrast, operates under constant temperature (~60°C) within a closed-tube format, integrating specific primers, thermostable polymerase, and a luminescent detection system to achieve real-time amplification and readout within 60–75 minutes (Zhang et al., 2020). Compared with RPA, AmpFire provides broader multiplexing capacity and fluorescence-based quantification, enabling simultaneous identification of up to 15 high-risk HPV types with detection limits as low as 2–20 copies per reaction under optimized conditions (Paytubi et al., 2022). Another comparative study with the Cobas 4800 assay (Zhang et al., 2023) reported that AmpFire exhibited comparable sensitivity in detecting high-risk HPV genotypes. Moreover, the AmpFire system can be directly applied to unprocessed clinical samples and supports high-throughput testing, making it particularly suitable for large-scale screening and use in resource-limited settings. However, its quantitative accuracy may vary across sample matrices, and the system lacks the sequence-level resolution of NGS.

Despite their advantages, IATs still face challenges such as complex primer design (especially for LAMP), variable performance across different sample types (e.g., urine, plasma, crude lysates), and differing sensitivity to inhibitory substances. To enhance accuracy and adaptability, these methods are increasingly integrated with specific readout systems like CRISPR/Cas detection or lateral flow assays, forming a dual-layer strategy that combines rapid amplification with precise molecular recognition for improved POCT performance. Collectively, isothermal platforms such as RPA, LAMP, and AmpFire demonstrate complementary strengths—RPA enables ultra-fast amplification with minimal temperature control, LAMP offers efficient amplification with simple visual detection, and AmpFire bridges laboratory and field use through a closed-tube, real-time luminescent system. Together, they provide flexible, sensitive, and cost-effective alternatives to PCR, holding strong potential for next-generation HPV diagnostics, especially in resource-limited and decentralized screening settings. Specific features are shown in the **Table 4**.

**Table 4:** Comparison of isothermal amplification techniques and conventional PCR for HPV detection (Rohatensky et al., 2018; Zhang et al., 2020; Liu et al., 2024).

Characteristics	RPA	AmpFire	LAMP	Conventional PCR/qPCR
Temperature	37–42°C	60–65°C	60–65°C	55–95°C (thermal cycling)
Primer Design	Single pair of primers	Two primer pairs (per target)	Six primers (FIP, BIP, F3, B3, LF, LB)	One primer pair
Amplification Time	15–30 minutes	10–15 minutes	30–60 minutes	1-2h
Sensitivity	1–10 copies/μL (HPV16/18/31/33/35/45)	Comparable to Cobas/NGS for CIN2+/3+	10–100 copies/μL	10–100 copies/reaction
Key Advantage	Ambient temperature compatibility; fast; minimal equipment	Fastest turnaround;Closed-tube real-time readout; adaptable to automation	Rapid, visual detection; high amplification efficiency; field-deployable	High sensitivity and specificity; quantitative

Collectively, emerging technologies such as ddPCR, NGS, IAT, and CRISPR/Cas12a are reshaping the landscape of HPV diagnostics by offering higher sensitivity, multiplexing capability, and greater adaptability to resource-limited settings. These methods enable more precise viral quantification, efficient detection of low-copy HPV DNA, and faster, equipment-sparing workflows, thus addressing many limitations of traditional techniques (Liu et al., 2024; Yin et al., 2024). Particularly, the integration of CRISPR/Cas systems with isothermal amplification methods presents a promising direction for developing rapid, low-cost, and user-friendly diagnostic tools. With further clinical validation and technical optimization, these innovations are poised to significantly improve the accuracy, accessibility, and scalability of HPV screening worldwide.

### 2.3 The role of artificial intelligence and computational biology in HPV diagnostics

Recently, artificial intelligence (AI) and computational biology are increasingly shaping the future of HPV diagnostics by enhancing the interpretation of complex molecular datasets generated from NGS, ddPCR, CRISPR-based assays, and liquid biopsy platforms. Through advanced machine learning and data fusion, AI enables the identification of subtle biomarker patterns that are often missed by traditional statistical analyses.

For example, recent AI-driven genomic analysis advances have enabled automated interpretation of viral–host interactions. The DeepHPV model exemplifies this: using an attention-based deep learning framework trained on 3,608 known sites, it achieved an AUROC of 0.84 in predicting HPV integration (Tian et al., 2021). What’s more, HPV-RNA-Seq, which combines viral and host transcriptomic profiles, employed a machine learning classifier to distinguish HSIL from low-grade or normal cytology samples, yielding a sensitivity of 93% and specificity of 88% for CIN2+ prediction (Faillace Thiesen et al., 2025). In addition, AI-enabled methylation-based models such as the SMART-HPV platform demonstrated high predictive performance (AUC = 0.95) in stratifying cervical cancer risk by integrating HPV genotyping with epigenetic signatures (Dong et al., 2025). These findings underscore the potential of AI in multi-parametric biomarker analysis, moving beyond single-molecule detection toward integrative, individualized HPV risk assessment.

Beyond viral DNA abundance, advanced cfDNA analyses including fragmentomics and methylation profiling provide orthogonal signals that AI models can exploit to improve both sensitivity and tissue-of-origin specificity. Reviews and recent studies show that fragment-level features and methylation patterns, when learned by ML models, markedly enhance early-stage detection performance across cancers and are directly applicable to HPV-driven malignancies when combined with viral markers (Tsui et al., 2025). Similarly, circulating miRNA panels and EV-RNA profiles have been incorporated into classifiers that stratify precancerous lesions and predict outcomes; machine learning improves signal extraction from noisy miRNA data and boosts predictive accuracy (Kniazeva et al., 2023; Xin et al., 2024).

In summary, the integration of AI with molecular HPV diagnostics represents a key step toward precision prevention and early intervention, transforming current laboratory-based assays into intelligent, patient-centered diagnostic ecosystems. To fully translate these models into clinical use, however, several challenges must be addressed. Large-scale, multi-center validation cohorts are needed to ensure generalizability across diverse populations. Moreover, standardized data pipelines, longitudinal sampling, and explainable AI models are critical for integrating cfDNA or EV-based HPV signatures into clinical workflows. Ultimately, coupling AI with liquid biopsy biomarkers could enable real-time monitoring of vaccine efficacy, treatment response, and minimal residual disease, paving the way for precision HPV management and early cancer prevention.

## 3 Innovations in sampling approaches

While advancements in detection technologies have greatly improved the sensitivity and specificity of HPV screening, the effectiveness of these methods is equally dependent on the quality, accessibility, and acceptability of the collected specimens. Conventional cervical sampling, though widely used, often involves invasive procedures that may deter participation, especially in low-resource or culturally sensitive settings. To address these limitations and expand the reach of HPV screening, recent research has increasingly focused on innovative, non-invasive sampling approaches—including liquid biopsy and self-sampling—that offer greater convenience, patient compliance, and scalability. This section explores the progress and potential of these novel sampling strategies in the context of HPV detection.

### 3.1 Liquid biopsy

Liquid biopsy has emerged as a transformative approach for HPV detection, leveraging circulating biomarkers—circulating tumor cells (CTCs), cell-free nucleic acids (cfNAs), or extracellular vesicles (EVs)—to enable non-invasive, real-time monitoring of HPV-related diseases (Tanzi et al., 2013; Cai et al., 2015; Zheng et al., 2019; Siravegna et al., 2022). This section critically evaluates these three modalities, focusing on their clinical utility, technical challenges, and future potential.

#### 3.1.1 Circulating tumour cell assay

CTCs are tumor-derived cells shed into peripheral blood. Detection relies on epithelial markers (e.g., EpCAM) via platforms like CellSearch^®^ or microfluidics (Heitzer et al., 2013; Haber and Velculescu, 2014).

However, studies have found that CTCs are rare (<10 cells/mL in metastatic cancer) and often undetectable in early-stage HPV-related cancers (Heitzer et al., 2013; Haber and Velculescu, 2014). Additionally, epithelial-mesenchymal transition (EMT) reduces EpCAM expression, lowering detection accuracy (Takakura et al., 2018). In conclusion, the features of low abundance and EMT-driven marker loss limit utility in early screening, and high cost and technical complexity hinder widespread adoption. Therefore, CTCs are more suited for metastatic monitoring than early HPV detection. In contrast, the use of exosomes or circulating free nucleic acids for HPV detection offers advantages such as non-invasiveness, convenience, and high patient acceptability (Ducancelle et al., 2015; Wang et al., 2020), particularly suitable for large-scale screening, making it a more promising detection method.

#### 3.1.2 Circulating free nucleic acid test

Cell-free nucleic acids (cfNAs), including HPV DNA, RNA, and miRNAs released into biofluids (e.g., blood, urine), represent a non-invasive alternative for HPV detection and genotyping (Cai et al., 2015). PCR-based methods such as qPCR and ddPCR amplify these fragments, enabling direct assessment of HPV infection and transcriptional activity.

In a study employing Sanger sequencing to detect hr-HPV in menstrual blood (MB), the detection sensitivity achieved 97.7%, and certain MB samples were able to identify genotypes that were missed by traditional tests (Zhang et al., 2021). Furthermore, urine samples have demonstrated good reliability in HPV genotyping. Ducancelle et al.’s research proved that urine samples can detect 14 hr-HPV types and effectively differentiate HPV16 and 18 from the other 12 high-risk types, providing clinicians with more precise information on infection types (Ducancelle et al., 2015). These findings indicate that urine testing has strong discriminatory power in HPV genotyping, offering reliable support for precise diagnosis. Another significant advantage is the convenience of sample collection. The relevant researches has reported that participation rates for urine/blood sampling (13.7–15.4%) exceeded those for cervical smears (10%) (Ducancelle et al., 2015; Lefeuvre et al., 2020), and the diagnostic cost-effectiveness reduced by 36–38% and shortened turnaround time by 26 days (Siravegna et al., 2022).

However, despite the high sensitivity and specificity of liquid biopsy in HPV DNA detection, in different studies, the HPV DNA concentration in urine fluctuates widely, leading to inconsistent and variable sensitivity (4.2–83.9%) (Ducancelle et al., 2015; Asciutto et al., 2017), these discrepancies may be attributed to the sample storage conditions and processing protocols. In a word, while cfNA testing offers accessible and cost-efficient screening, standardization of protocols is critical to address variability and enhance reliability in clinical practice.

#### 3.1.3 Extracellular vesicles assay

EVs are small membrane-bound vesicles secreted by cells, typically ranging in diameter from 30 to 150 nanometers (nm), and containing a variety of intracellular molecules such as proteins, lipids, nucleic acids (DNA, RNA) and miRNAs (e.g., let-7d-3p, miR-30d-5p). The acquisition methods involves ultracentrifugation or commercial kits (e.g., ExoQuick^®^) (Zheng et al., 2019; Wang et al., 2020; Kumar et al., 2024).

EVs offer convenient collection methods and are abundant in quantity, with up to one billion particles per milliliter of bodily fluid, providing sufficient material for detection (Cai et al., 2015). Furthermore, EVs carry information from viable cells and can reveal early signals of HPV infection. Wang et al. found that EVs extracted from the saliva of patients with HPV-associated oropharyngeal cancer could accurately detect HPV16 DNA, with a consistency rate of up to 80% when compared to tissue biopsy results (Wang et al., 2020). Additionally, Zheng et al.’s research demonstrated that the combination of miRNAs (let-7d-3p and miR-30d-5p) in plasma exosomes had high accuracy (AUC = 0.922) in distinguishing between CIN 1 and CIN 2+ patients, outperforming traditional cytology (AUC = 0.766) (Zheng et al., 2019). Studies have shown that, due to their unique lipid bilayer structure, exosomes exhibit extreme biological stability. This stability ensures that the quality of exosomes remains reliable even after long-term storage, allowing for long-term tracking and monitoring, and bringing great convenience to subsequent isolation and detection work (Zheng et al., 2019; Yu et al., 2021).

However, the isolation and purification of exosomes still face challenges. Factors such as the type of body fluid, sampling time, and storage conditions can affect the stability of the results (Li et al., 2017; Wang et al., 2020). Additionally, there are significant differences in the expression levels of miRNAs across different bodily fluids, which may impact the accurate reflection of the tumor’s actual condition. One study comparing the content of seven miRNAs in exosomes from plasma versus cervical cancer tissue and adjacent tissue in the same validation system found that the ratios of these miRNAs in tissue and exosomes were inconsistent, with a Spearman correlation coefficient of only 0.321 (Zheng et al., 2019). Despite these challenges, exosomes show great potential for the early diagnosis of HPV-related cancers. Future research needs to optimize their isolation and purification techniques and conduct large-scale clinical validation to improve the accuracy and stability of detection.

The integration of liquid biopsy modalities like cfDNA and EVs is refining HPV screening, enhancing early detection and paving the way for personalized management. Collectively, these liquid biopsy modalities offer complementary insights into HPV-associated disease dynamics. Beyond early detection, their longitudinal monitoring capacity may provide an opportunity to assess therapeutic efficacy and vaccine-induced viral clearance. For example, in cervical cancer, recent prospective data show that serial quantification of HPV cfDNA (during and after chemoradiation) closely tracks treatment response; patients who achieve early cfDNA clearance have significantly better recurrence-free survival than those with persistent cfDNA (Han et al., 2024; Seo et al., 2024). Moreover, detection of HPV E6/E6*I transcripts within EVs supports their potential in monitoring viral transcriptional activity under treatment (Bhat et al., 2022). Looking forward, integrating longitudinal liquid biopsy with vaccination or therapeutic interventions may enable real-time assessment of vaccine efficacy and residual disease. To achieve clinical translation, multicenter validation and standardized workflows will be essential to define sensitivity thresholds and temporal sampling strategies.

### 3.2 Self-sampling test

HPV self-sampling involves the use of biological specimens (such as vaginal secretions) collected by patients themselves. These specimens are then analyzed for HPV nucleic acids through molecular biology techniques, including PCR or real-time fluorescent quantitative PCR (Haguenoer et al., 2014; Auvinen et al., 2022). This approach eliminates the need for clinician-collected cervical samples, addressing barriers to screening accessibility and patient discomfort.

Studies have demonstrated that self-sampling can significantly increase participation rates by 20–30% in cervical cancer screening among underscreened populations, particularly in resource-limited settings (Haguenoer et al., 2014; Gustavsson et al., 2018). In terms of diagnostic accuracy, self-sampling has been shown to match clinician-collected samples in both sensitivity (96%) and specificity (100%) for detecting CIN2+ lesions (Polman et al., 2019). Moreover, self-sampling proves to be cost-effective, reducing screening costs by 25–30% and shortening turnaround time by 17 days (Su et al., 2023). This facilitates quicker test results and improves screening efficiency.

However, despite the high accuracy demonstrated by self-collected samples in HPV testing, sample quality remains a potential concern. Studies have shown that 5–10% of self-samples require retesting due to insufficient cellularity or degradation (Haguenoer et al., 2014; Polman et al., 2019), adding complexity and time costs to the testing process. Additionally, the pre-analytical variables (e.g., storage temperature, transport protocols) may impact reproducibility. For example, in the study by Andersen et al., six women (6.5%) had HPV-positive self-collected samples but HPV-negative clinician-collected samples (Andersen et al., 2022), which may impact the accurate interpretation of test results. Furthermore, there is the problem of subtype discordance, studies have shown that a few self-collected or clinician-collected samples tested positive for HPV types (such as HPV30, 53, 67, and 70) using NGS methods, but these types were not detected by Cobas4800 and CLART (Andersen et al., 2022).

In summary, innovative sampling approaches—such as liquid biopsy and self-collection—offer promising alternatives to conventional cervical sampling by enabling non-invasive, repeatable, and more acceptable testing strategies. Liquid biopsy, through the detection of HPV nucleic acids or exosome-derived markers in blood, urine, or saliva, demonstrates high sensitivity and genotyping consistency with cervical samples, supporting its potential for early diagnosis and population-scale screening. Meanwhile, self-sampling improves participation rates and overcomes cultural or logistical barriers to clinic-based screening. Despite ongoing technical challenges in sample processing and standardization, the integration of these user-friendly sampling methods with emerging detection platforms is expected to significantly enhance the accessibility and precision of HPV testing, accelerating the shift toward more inclusive and patient-centered screening programs.

## 3 Conclusion

Currently, despite the widespread clinical application of HPV detection methods such as HC2, Cervista, BD, PapilloCheck, and Abbott, they still face numerous challenges. These include the invasiveness of sampling techniques, instability in detection sensitivity and specificity, operational complexity, high costs, and issues related to patient acceptance. These challenges have, to some extent, constrained the popularization and application of these methods. With technological advancements, emerging HPV detection techniques such as NGS, CRISPR/Cas12a, and liquid biopsy have the potential to revolutionize HPV diagnostic technology by offering more precise and non-invasive detection methods. These emerging techniques are poised to replace traditional methods, particularly due to their greater potential in terms of convenience, accuracy, patient experience, and acceptance.

In future research, the development of HPV detection technologies should closely focus on the following aspects: Firstly, enhancing the sensitivity and specificity of HPV detection is a crucial goal, necessitating further optimization of existing technologies. This includes refining PCR primer design, optimizing guide RNA sequences for the CRISPR/Cas system, and developing more efficient methods for exosome isolation to reduce the occurrence of false positives and false negatives. Secondly, promoting the popularization of non-invasive detection technologies is an important direction. Emphasis should be placed on developing HPV nucleic acid detection methods based on blood, urine, and saliva, thereby reducing the reliance on invasive sampling, enhancing patient acceptance, and increasing screening coverage. Thirdly, achieving precise detection of HPV genotyping and viral load is a key focus of future research. By integrating high-resolution technologies such as ddPCR and NGS, accurate identification of hr-HPV subtypes and their viral loads can be achieved, providing a scientific basis for risk assessment and personalized treatment. Fourthly, the development of low-cost, portable HPV detection devices is a vital pathway for the popularization of screening technology, especially in resource-limited areas. The research and development of simple-to-operate, low-cost detection tools are needed to meet the demands of large-scale screening. Fifthly, exploring novel biomarkers is an important means to improve detection accuracy. For instance, by analyzing HPV nucleic acids or protein biomarkers in exosomes, as well as HPV integration sites in ctDNA, more reliable evidence for early diagnosis can be provided. Lastly, the integration of artificial intelligence and big data technology will drive the intelligent development of HPV detection. By establishing risk prediction models for HPV infection and automated analysis platforms, screening strategies can be optimized, and detection efficiency can be improved. Through interdisciplinary collaboration and technological innovation, emerging HPV detection methods are expected to significantly enhance the accuracy and feasibility of early cervical cancer screening, thereby markedly reducing the incidence and mortality rates of HPV-related diseases. In the future, with the widespread application and continuous improvement of these technologies, HPV detection will become more precise, convenient, and accessible, making significant contributions to achieving global HPV-related disease prevention and control goals.

## Glossary

HC2: Hybrid Capture 2
BD Onclarity: BD Onclarity HPV Assay
AHPV: APTIMA HPV E6/E7 mRNA Test
PapilloCheck: PapilloCheck HPV Test
Abbott: Abbott Real-Time High-Risk HPV Test
NGS: Next-Generation Sequencing
ddPCR: Droplet Digital PCR
IAT: Isothermal Amplification Technology
LAMP: Loop-mediated Isothermal Amplification
RPA: Recombinase Polymerase Amplification
hr-HPV: high-risk Human Papillomavirus
EVs: Extracellular Vesicles
CIN: Cervical Intraepithelial Neoplasia
SMRT: Single-Molecule Real-Time
SERS: Surface-Enhanced Raman Scattering
LSPR: Localized Surface Plasmon Resonance
ctDNA: Circulating Tumor DNA
cfNAs: Circulating Free Nucleic Acids
CTCs: Circulating Tumour Cells
EMT: Epithelial-Mesenchymal Transition
FFPE: Formalin-Fixed Paraffin-Embedded
LBC: Liquid-Based Cytology
ICP-MS: Inductively Coupled Plasma Mass Spectrometry
CRISPR: Clustered Regularly Interspaced Short Palindromic Repeats
gRNA: Guide RNA
crRNAs: CRISPR RNAs
cfDNA: cell-free DNA
POCT: point-of-care testing
AI: artificial intelligence
sgRNAs: single-guide RNAs
ctPCR: CRISPR-typing PCR
CARP: Cas9/sgRNA-associated reverse PCR
